# Intestinal schistosomiasis masquerading as intestinal polyps

**DOI:** 10.1186/s12879-021-06125-4

**Published:** 2021-05-08

**Authors:** Xiu Li Zhu, Ji Zhong Song, Wen Yong Yu, Li Qun Hua, Ming Li Zhang

**Affiliations:** Department of Gastroenterology,The First Affiliated Hospital of USTC, Division of Life Sciences and Medicine, University of Science and Technology of China, Hefei, Anhui 230001 PR China

**Keywords:** Intestinal schistosomiasis, Intestinal polyps, Schistosomal appendicitis

## Abstract

**Background:**

Schistosomiasis is very common in the southern part of the Yangtze River Basin in China. It is mainly manifested as appendicitis, ulcers, hematomas, and thickening of the intestinal tract. Schistosomiasis of the appendix is rare, mainly manifested as appendicitis, which is easy to be misdiagnosed.

**Case presentation:**

Here we report a rare case of a Chinese female whose intestinal mass manifested as intestinal polyps and was eventually diagnosed pathologically as schistosomiasis infection (appendix schistosomiasis). So far, there are rare relevant cases reported.

**Conclusions:**

Intestinal schistosomiasis is easily misdiagnosed, and appendix schistosomiasis is rare. The final diagnosis requires pathology, especially surgical pathology.

## Background

Schistosomiasis is a parasitic disease caused by the trematode of the genus *Schistosoma*. In the public health sense, it is second only to malaria, infecting more than 200 million people worldwide, causing severe consequences of 20 million people and 100,000 deaths each year [1, 2]. The disease is mainly manifested in the liver, intestine, urogenital tract, and blood system. It mainly manifests as liver fibrosis in China. Schistosomiasis is very common in the southern Yangtze River basin of China, and such patients are under a history of living at the source of the schistosomiasis epidemic. Intestinal schistosomiasis mainly manifests as appendicitis, ulcers, hematomas, and thickening of the intestine [3]. Among them, schistosomiasis infection of the appendix is prone to gangrene and perforation, but a mass in the appendix is rare. The following case describes a female whose intestinal mass appeared as intestinal polyps and was eventually diagnosed with a schistosomiasis infection of the intestine and appendix by pathology. To the best of our knowledge, there are rare relevant cases reported in the Chinese and English literature.

## Case presentation

**Fig. 1:**
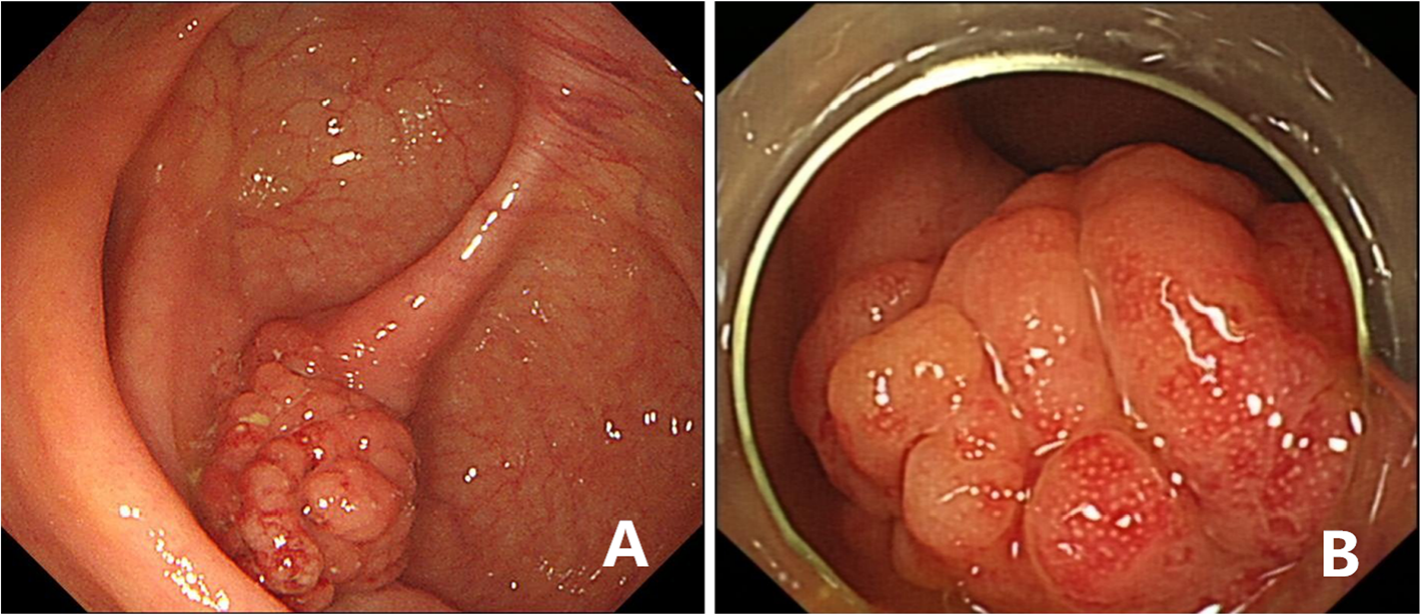
Colonoscopy revealed a polypoid-like hump of approximately 2.5 cm × 3.0 cm behind the the ileocecal valve (**a**), with a thickened superficial duct opening, congestion, and exudation of purulent discharge (**b**)

**Fig. 2:**
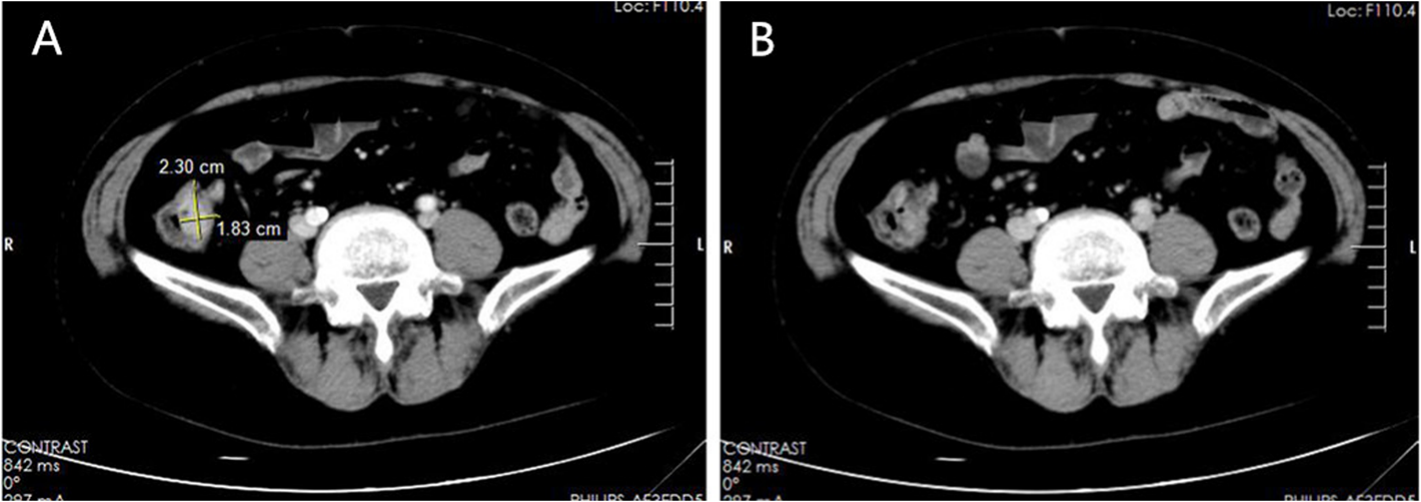
CT scan showed a soft tissue approximately 2.3 cm × 1.8 cm in the ileocecal area with irregular borders (**b**), and moderately continuous enhancement after enhancement (**a**)

A 65-year-old female from Chaohu, Anhui, China attended our hospital, complaining of slimy stool with mucus for more than 10 years. She had no history of schistosomiasis epidemiology and exposure. She had no family history of colon cancer. The patient did not have any organomegaly on examination. Her blood tests (blood routine, erythrocyte sedimentation rate, C-reactive protein, tumor marker CA199, CEA, CA125, AFP) and stool tests were normal. Colonoscopy revealed a polypoid-like hump of approximately 2.5 cm × 3.0 cm behind the ileocecal valve, with a thickened superficial duct opening, congestion, and exudation of purulent discharge. The surrounding mucosa was hyperemic, and the appendix was not visible **(**Fig. 1**)**, the remaining colonic mucosa had no lesions or inflammation. The histopathology of the ileocecal area showed severe chronic inflammation and lymphoid follicular hyperplasia. The entire abdominal CT scan before the colonoscopy showed a soft tissue approximately 2.3 cm × 1.8 cm in the ileocecal area with irregular borders, and moderately continuous enhancement after enhancement **(**Fig. 2**)**.

We initially diagnosed the bulge of the ileocecal as intestinal polyps and prepared to undergo endoscopic submucosal dissection (ESD) surgical resection, however, during the operation we found that the lesions may not be completely cleared, the patient finally decided to undergo surgery. In this colonoscopy, we performed the pathological examination of the same part, and the results were similar to the previous results. Laparoscopy showed no ascites in the abdominal cavity, rich omentum tissue, no metastasis in the liver and peritoneum, and the ileocecum and ascending colon omentum were wrapped inside the abdominal wall. The tumor was located at the opening of the appendix in the ileocecal region, which was also the root of the appendix, without invading the serosa.

**Fig. 3:**
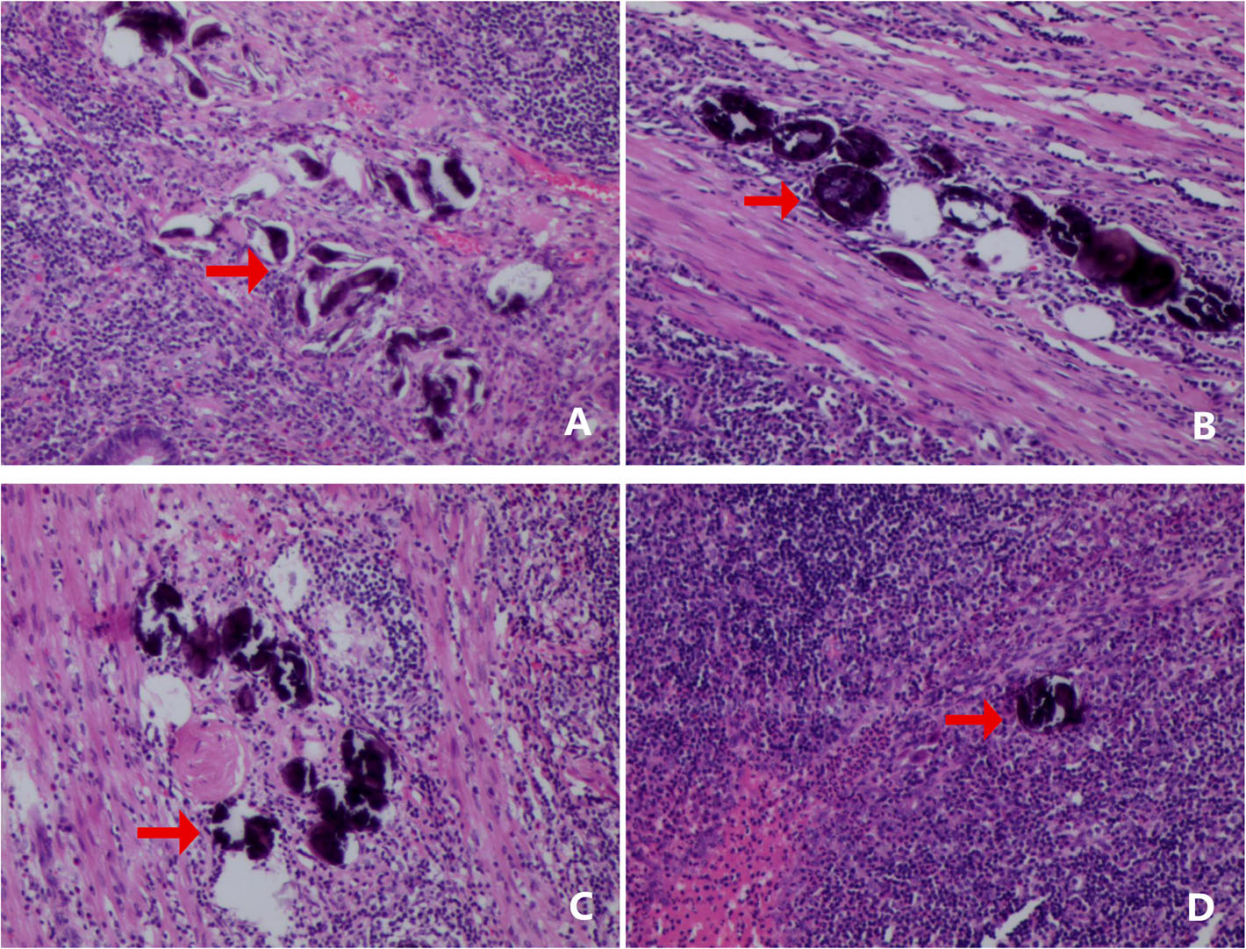
There was a large amount of schistosomiasis eggs deposited in the ileocecal region (**a**, **b**) and the appendix (**c**, **d**), with a lots of lymphocytes, plasma cells, eosinophils, and neutrophils infiltrated. The red arrow marks the eggs

The tumor markers of the patient were normal, but the malignant tumor could not be ruled out. The surgeon and his family decided to completely remove the right half colon after consultation. Histopathology clearly showed that there were a large number of schistosome eggs deposited in the ileocecal region and the appendix, with a lot of lymphocytes, plasma cells, eosinophils, and neutrophils infiltrated, lymphoid tissue proliferation, and lymphoid follicle formation **(**Fig. 3). We carried out a total of three pathological tests and finally diagnosed with intestinal schistosomiasis (ileucopia, appendix).

## Discussion and conclusions

Schistosomiasis is still prevalent in China. An epidemiological survey in the Chaohu area of Anhui Province shows that schistosomiasis is still prevalent there [4–6]. Oncomelania hupensis is the intermediate host of schistosomiasis. Research data showed that (1) a total of 314 residents were detected by indirect hemagglutination assay (IHA), but there were no positives; (2) a total of 302 mobile population were detected by IHA, and the positive rate of antibody was 1.32%; (3) 30 individuals were examined by stool tests, and the positive rate was 20% [6]. The patient is from Chaohu, Anhui Province, but she has no relevant epidemiological history, we can’t rule out the possibility of unknown potential infection.

The diagnosis of intestinal schistosomiasis is very difficult, and it is almost impossible to confirm the diagnosis before surgery. It may help to diagnose by asking for the previous history. The gold standard for the diagnosis of an ongoing schistosome infection has for decades been the detection of eggs in a fecal smear. However, the positive rate of fecal worm eggs is very low, histopathology and serology can also help diagnosis [7]. The low positive rate of fecal eggs may be related to fecal sampling, specimen preservation and time, etc. The final diagnosis of our patient was the discovery of schistosome eggs in surgical specimens.

Schistosome eggs can invade many parts of the gastrointestinal tract, urinary tract, etc., but mainly cause lesions in the liver, spleen, and colon, causing tissue fibrosis or bleeding [8]. Intestinal schistosomiasis is classified into three types of enteritis acute, chronic, and mixed type, the latter was an important and independent type. The main mechanism of schistosomiasis is that cercariae invade the human body and invade the portal vein system through blood circulation. Eggs fall off with necrotic tissue, enter the intestinal cavity, and deposit in the appendix cavity. Mature worm eggs containing hairy maggots release soluble antigens through the pores of the eggshell and activate the host’s immune system and cause a local immune response.

Acute patients present with immune complex disease, while chronic patients present with T lymphocyte-mediated delayed allergies, which may be accompanied by the deposition of antigen-antibody complexes to form eosinophilic granuloma. With the degeneration, necrosis, and calcification of worm eggs, infiltrating cells were replaced by monocytes and fibroblasts to form chronic granulomas [9].

The disease was easily misdiagnosed as colon cancer, ulcerative colitis, and intestinal tuberculosis. Isolated schistosomiasis infections are rare. Colonoscopy findings and repeated biopsy from the suspected lesions were essential for getting the correct diagnosis, but the positive rate was low, sometimes surgery was needed to confirm the diagnosis. The prevention and control of schistosomiasis are very important, and medical staff needs to strengthen their understanding of schistosomiasis to avoid missing diagnosis.

## Data Availability

The datasets used and/or analysed during the current case reports are available from the corresponding author on reasonable request.

## Abbreviation

ESD: Endoscopic submucosal dissection
IHA: Indirect hemagglutination assay
